# Fellow-Workers and Their Doings

**Published:** 1914-07-25

**Authors:** 


### ROUND THE WORLD.

### Fellow-Workers and their Doings.

[The Editor Invites Early Information and Contributions Marked "Fellow-Workers."]

Sir William Watson Cheyne, M.B., F.R.C.S., F.R.S., is to be congratulated on his election to the presidency of the Royal College of Surgeons of England. It is generally considered that it was upon Sir Watson Cheyne that the mantle of the late Lord Lister fell when that remarkable exponent of surgery retired from active work, for they were for some years closely associated in work at King's College Hospital, where the new President is a member of the surgical staff and professor of clinical surgery. It will be remembered that Sir Watson Cheyne, who was created a baronet in 1908, went out to South Africa as consulting surgeon to the forces at the time of the last Boer War; among other appointments, he holds that of honorary surgeon in ordinary to His Majesty.

Sir Francis H. B. Champneys, who has just retired from the presidency of the Royal Society of Medicine, has been a very popular director of that Association's work. Best known, of course, in connection with obstetrics and gynæcology—to the profession as consulting physician accoucheur to St. Bartholomew's, and to the general public as chairman of the Central Midwives' Board—Sir Francis has done much valuable work in his special subjects, apart from which his intimates recognise in him a widely-read student of literature and general medicine. Qualifying M.B. Oxon., M.R.C.S. Eng. in 1875, taking the M.D. in 1888, and becoming elected F.R.C.P. Lond. in 1882, Sir Francis Champneys has had a very distinguished career in London; he was created a baronet in 1910.

His successor in the presidential chair of the Royal Society of Medicine, Dr. Frederick Taylor, is also a popular leader of the medical profession. Having been associated for many years with Guy's Hospital, in connection with which he now holds the post of consulting physician, Dr. Taylor has also become well known to many medical men as examiner in medicine at several universities and colleges. He has also held high office at the Royal College of Physicians, including that of senior censor. Dr. Taylor is also the author of numerous writings, including a popular text-book on practical medicine, which has lately reached its tenth edition.

Of the two junior surgeons who have just been appointed surgical registrars and demonstrators of anatomy at Guy's Hospital Medical School, Mr. G. T. Mullally was awarded the Treasurer's gold medal in clinical surgery in 1911. Since then he has been doing a good deal of official teaching, as well as giving anæsthetics in the dental department; while Mr. R. C. Ozanne distinguished himself by taking first-class honours in physiology at Oxford, where he subsequently took the degrees of M.A., M.B., B.Ch. Since qualifying he has held the post of house surgeon at "Guy's." Mr. Mullally, who is an M.B., B.S. Lond., has had some experience of medical journalism as editor of the *Guy's Hospital Gazette*.

The new assistant dental surgeon at the Royal Dental Hospital, Mr. F. St. Jermain Steadman, has been for some time attached to the National Dental College. In addition to his dental qualifications, he holds the "conjoint" diploma, having studied medicine both at the London and Charing Cross Hospitals. Mr. Steadman holds several other institutional appointments in London, and has written a good deal on dental pathology.

Hammersmith's new medical officer of health, Dr. J. B. Howell, is transferring his work from Stepney, where he has been assistant medical officer of health and bacteriologist. He qualified M.R.C.S., L.R.C.P., from University College Hospital in 1905, and then, interesting himself in State medicine, became a D.P.H. some three years ago. As a student he won a gold medal in midwifery and gynæcology and the Liston gold medal in surgery. Dr. Howell is a member of the Royal Sanitary Institute, and was at one time on the staff of Hanwell Asylum.

Dr. J. W. Astley Cooper, who opened the debate on the proposed legislation for inebriates before the Society of Inebriety last week (July 14, 1914), is well known as an authority on this special subject, and is superintendent of an institution for the cure of drunkards. Of late years Dr. Astley Cooper has been an advocate of the use of psychotherapeutic methods in conjunction with routine physical measures in the treatment of alcoholism and the drug habit, his results in this particular field having been very instructive. He is the author of a work on "Pathological Inebriety, Its Causes and Treatment."

The new chief assistant in the electrical department at St. Bartholomew's Hospital, Mr. Alastair MacGregor, has been working for some time in the electrical department at the West London Hospital. He qualified M.B., C.M. Edin., in 1885, and some years later became an M.D. of the same university.

Mr. Gerald Stanley, who has just been appointed assistant surgeon to the *Dreadnought* Hospital at Greenwich, obtained the high surgical qualifications of M.S. Lond. and F.R.C.S. Eng. two years ago. He is a distinguished student of St. Bartholomew's Hospital, where he gained the Bentley Prize, the Brackenbury Surgical Scholarship, and the Walsham Prize in Pathology. At the present time he holds the posts of demonstrator in anatomy and chief assistant in the surgical out-patient department there. Mr. Stanley is one of those junior men of whom great things may be predicted.

Any work that will enable the practitioner to carry out clinical estimations with increased facility has obvious merits. Thus much interest attaches to a quick method of measuring sugar percentages which has been reported to the *British Medical Journal* (July 4, 1914) by Mr. G. C. Parnell, M.R.C.S. This method depends on a colour test, certain standard shades being arrived at by test reductions of sugar solutions. In practice the fluid to be tested is boiled with liquor potassæ, and the result in colour compared with the standard test colours which, for purposes of convenience, are provided as tinted glasses. Mr. Parnell, who is an old "Bart.'s" man in practice at Forest Hill, holds the appointment of consulting surgeon to the Home for Sick Children in Lower Sydenham.

